# The Interplay of Molecular Factors and Morphology in Human Placental Development and Implantation

**DOI:** 10.3390/biomedicines12122908

**Published:** 2024-12-20

**Authors:** Ioana Vornic, Victor Buciu, Cristian George Furau, Flavia Zara, Dorin Novacescu, Alina Cristina Barb, Alin Adrian Cumpanas, Silviu Constantin Latcu, Ioan Sas, Denis Serban, Talida Georgiana Cut, Cristina Stefania Dumitru

**Affiliations:** 1 Doctoral School, Department Medicine, “Vasile Goldiș” Western University of Arad, Liviu Rebreanu Street, No. 86, 310414 Arad, Romania; ioana_vornic@yahoo.com; 2 Discipline of Gynecology, Department Medicine, Vasile Goldiş Western University, Liviu Rebreanu Boulevard, No. 86, 310414 Arad, Romania; cristianfurau@gmail.com; 3 Doctoral School, Victor Babes University of Medicine and Pharmacy Timisoara, E. Murgu Square, No. 2, 300041 Timisoara, Romania; silviu.latcu@umft.ro; 4 Department II of Microscopic Morphology, Victor Babes University of Medicine and Pharmacy Timisoara, E. Murgu Square, No. 2, 300041 Timisoara, Romania; flavia.zara@umft.ro (F.Z.); novacescu.dorin@umft.ro (D.N.); toma.alina@umft.ro (A.C.B.); cristina-stefania.dumitru@umft.ro (C.S.D.); 5 Department XV, Discipline of Urology, Victor Babes University of Medicine and Pharmacy Timisoara, E. Murgu Square, No. 2, 300041 Timisoara, Romania; cumpanas.alin@umft.ro; 6 Department XII, Discipline of Gynecology and Obstetrics, Victor Babes University of Medicine and Pharmacy Timisoara, E. Murgu Square, No. 2, 300041 Timisoara, Romania; sas.ioan@umft.ro (I.S.); denis.serban@umft.ro (D.S.); 7 Department XIII, Discipline of Infectious Diseases, Victor Babes University of Medicine and Pharmacy Timisoara, E. Murgu Square, No. 2, 300041 Timisoara, Romania; talida.cut@umft.ro; 8 Center for Ethics in Human Genetic Identifications, Victor Babes University of Medicine and Pharmacy Timisoara, E. Murgu Square, No. 2, 300041 Timisoara, Romania

**Keywords:** placental development, morphology, fetal health, organogenesis, molecular pathways, VEGF, maternal-fetal interface

## Abstract

The placenta is a vital organ that supports fetal development by mediating nutrient and gas exchange, regulating immune tolerance, and maintaining hormonal balance. Its formation and function are tightly linked to the processes of embryo implantation and the establishment of a robust placental-uterine interface. Recent advances in molecular biology and histopathology have shed light on the key regulatory factors governing these processes, including trophoblast invasion, spiral artery remodeling, and the development of chorionic villi. This review integrates morphological and molecular perspectives on placental development, emphasizing the roles of cytokines, growth factors, and signaling pathways, such as VEGF and Notch signaling, in orchestrating implantation and placental formation. The intricate interplay between molecular regulation and morphological adaptations highlights the placenta’s critical role as a dynamic interface in pregnancy. This review synthesizes current findings to offer clinicians and researchers a comprehensive understanding of the placenta’s role in implantation, emphasizing its importance in maternal-fetal medicine. By integrating these insights, the review lays the groundwork for advancing diagnostic and therapeutic approaches that can enhance pregnancy outcomes and address related complications effectively.

## 1. Introduction

The placenta is a transitional organ whose components form in the first days of pregnancy. Structurally, it is a unique organ, being the only one formed from tissues that come from two different organisms. It ensures the nutrition, growth, and development of the product of conception and at the same time protects it from substances circulating in maternal blood through the maternal-fetal barrier. Under normal conditions, it is completely eliminated immediately after birth [1]. The development of the placental-uterine interface involves a dynamic interplay of molecular and cellular mechanisms, particularly trophoblast invasion and spiral artery remodeling. While most studies agree on the critical role of trophoblast cells in transforming spiral arteries into low-resistance vessels, controversies exist regarding the extent and timing of this transformation [2]. For instance, some research emphasizes the dual contributions of maternal immune cells, such as uterine natural killer (uNK) cells, in coordinating vascular remodeling, whereas other studies question their precise role, suggesting trophoblast-independent mechanisms may also contribute to spiral artery adaptation [3]. Furthermore, the degree of trophoblast invasion has been debated, with some evidence indicating incomplete remodeling may be a physiological variation rather than solely pathological, depending on gestational context [4,5]. For instance, variations in placental size, weight, and shape can be early indicators of potential complications, with significant implications for fetal outcomes [6]. Studies have shown that deviations in placental weight, particularly in relation to fetal weight, are associated with risks of adverse pregnancy outcomes, including growth restriction and preeclampsia. Additionally, specific structural characteristics, such as umbilical cord insertion site and the presence of accessory lobes, have been linked to fetal growth discrepancies and even perinatal mortality [7,8].

Despite these insights, many aspects of placental function and development remain partially understood. The formation and function of the placenta are intricately linked to embryo implantation, a highly coordinated process involving molecular and cellular interactions between the blastocyst and the maternal endometrium. Key processes, including trophoblast invasion, spiral artery remodeling, and the development of the villous tree, are regulated by an array of signaling molecules, such as vascular endothelial growth factor (VEGF), transforming growth factor-beta (TGF-β), and Notch signaling pathways [9]. These factors orchestrate the establishment of a functional placental-uterine interface, ensuring adequate maternal blood supply and immune tolerance. However, disruptions in these pathways can lead to abnormal implantation and placental development, contributing to pregnancy complications such as preeclampsia, intrauterine growth restriction (IUGR), and recurrent pregnancy loss [10,11].

This review aims to integrate the latest findings on the molecular mechanisms and morphological features of placental development, covering the key stages of organogenesis, structural adaptations, and clinical implications associated with placental abnormalities. By synthesizing current findings and identifying gaps in our understanding, we hope to highlight the importance of the placenta in maintaining fetal health and emphasize the need for continued research into this vital organ.

## 2. The Organogenesis of the Placenta

The human placenta is a remarkable organ that develops during pregnancy to support the growth and survival of the fetus. Its formation begins immediately after conception, involving a coordinated series of cellular and molecular interactions between maternal and embryonic tissues. These processes are driven by trophoblast differentiation, invasion, and the establishment of a functional uteroplacental interface [12], orchestrated by factors such as VEGF and transforming TGF-β [13]. Understanding the organogenesis of the placenta is important for appreciating its central role in nutrient and gas exchange, immune tolerance, and hormonal regulation. Furthermore, insights into the mechanisms of placental development provide valuable perspectives on the origins of pregnancy-related disorders such as preeclampsia and intrauterine growth restriction (IUGR), which are often linked to disruptions in early placental formation [14].

The journey of placental development begins at the earliest stages of embryogenesis, with fertilization occurring in the fallopian tube at the junction between the ampullary and isthmic portions. Following fertilization, the zygote undergoes rapid mitotic divisions, forming the morula by days 3–4 post-fertilization [12]. This solid ball of 12–16 cells transitions into a blastocyst, characterized by two distinct cell populations: the inner cell mass (embryoblast), which develops into the embryo proper, and the outer cell mass (trophoblast), the precursor to placental tissues. These early stages are governed by tightly regulated molecular signaling pathways, including Wnt and Notch signaling, which are critical for cellular differentiation and polarity. Understanding these processes is essential, as disruptions in early embryogenesis can impair trophoblast function and subsequent placental development [15].

The blastocyst enters the uterine cavity approximately 4–5 days post-fertilization, remaining free-floating for 1–2 days before initiating the implantation process. During this time, the protective zona pellucida surrounding the blastocyst degrades, allowing for direct interaction with the endometrial lining. Implantation occurs within a well-defined “implantation window”, during which the endometrium is optimally receptive due to progesterone- and estrogen-mediated changes. These hormonal signals upregulate key adhesion molecules, such as integrins and selectins, that facilitate attachment between the trophoblast and the maternal epithelium. While this window is crucial for implantation success, studies have debated the precise timing and molecular contributors, with some emphasizing the role of maternal cytokines over hormonal regulation (e.g., IL-11 vs. progesterone dominance) [16].

The role of cytokines is essential in the implantation process, orchestrating a complex interaction between the embryo and the maternal endometrium to facilitate successful implantation. Key cytokines involved include:

- Leukemia Inhibitory Factor (LIF): A member of the IL-6 cytokine family, LIF is essential for endometrial receptivity. It promotes the differentiation of endometrial cells and facilitates the adhesion of the blastocyst to the uterine lining. The study by Alzaidi Z. et al. showed that reduced LIF expression is associated with implantation failure and infertility [17].
- Interleukin-11 (IL-11): Also part of the IL-6 cytokine family, IL-11 is essential for decidualization—the transformation of endometrial stromal cells into specialized decidual cells that support embryo implantation. Deficiencies in IL-11 signaling can lead to impaired decidualization and subsequent implantation failures [17].
- Tumor Necrosis Factor-alpha (TNF-α): This cytokine has a dual role in implantation. At physiological levels, TNF-α contributes to tissue remodeling and immune regulation necessary for implantation. However, elevated levels of TNF-α are associated with inflammatory conditions that can disrupt implantation and are linked to pregnancy complications [18].

The coordinated expression and regulation of these cytokines ensure a conducive environment for the embryo to implant and develop. Disruptions in their levels or signaling pathways can lead to implantation failures and are implicated in various infertility conditions.

Implantation proceeds through three defined phases: pre-implantation, implantation proper, and post-implantation. In the pre-implantation phase, as the blastocyst nears the uterine wall, the endometrium transforms into decidual tissue through a process called decidualization. Progesterone, prostaglandins, and interleukins such as IL-11 orchestrate significant cellular enlargement, glycogen accumulation, and extracellular matrix remodeling within endometrial stromal cells. However, differing perspectives suggest that local maternal immune tolerance, particularly mediated by uNK cells, also plays a central role in preparing the endometrium for implantation. Abnormalities in decidualization are increasingly linked to recurrent implantation failure and early pregnancy loss, emphasizing the need for further studies on this interface [19].

The implantation proper phase begins around day 6–7 post-fertilization when the blastocyst attaches to the secretory endometrium. At this stage, trophoblast cells at the embryonic pole of the blastocyst rapidly proliferate and differentiate into two distinct layers: the syncytiotrophoblast, an outer multinucleated layer formed by the fusion of trophoblast cells, and the cytotrophoblast, an inner layer of mononuclear cells with high mitotic potential. The syncytiotrophoblast plays a pivotal role in the erosion of the endometrial epithelium, allowing the blastocyst to penetrate the underlying lamina propria [20]. This process, known as interstitial implantation, is unique to humans and higher primates. Molecular regulators such as matrix metalloproteinases (MMPs) facilitate the degradation of extracellular matrix components, ensuring successful invasion, while integrins mediate adhesion between trophoblast cells and the maternal tissue. Disruptions in this finely tuned process are implicated in implantation disorders, including ectopic pregnancies and early pregnancy loss [21].

As implantation progresses, the syncytiotrophoblast continues to expand and invade maternal tissues, promoting the formation of trophoblastic lacunae that later fill with maternal blood, establishing the foundation for maternal-fetal exchange. By day 11 post-fertilization, the blastocyst is completely embedded within the endometrium, with the implantation site covered by fibrin and cellular debris. This coverage is eventually replaced by regenerating surface epithelium, ensuring the integrity of the uterine lining. The post-implantation phase marks the formation of early placental structures, including primary villi and the initiation of the uteroplacental circulation. During this phase, human chorionic gonadotropin (hCG) secretion by the syncytiotrophoblast plays a critical role in maintaining the corpus luteum and sustaining progesterone production, which is vital for endometrial support and placental development [22]. Table 1 provides a comprehensive overview of the implantation stages, detailing the timing, key processes, structures formed, and associated hormonal changes essential for successful embryo implantation and early placental development.

As the placenta continues to develop, maternal tissues undergo extensive remodeling to support the demands of pregnancy. The functional endometrium transforms into the decidua, which differentiates into three distinct regions: the decidua basalis, forming the maternal component of the placenta; the decidua capsularis, covering the implantation site toward the uterine cavity; and the decidua parietalis, lining the remaining uterine mucosa. These regions collectively create an environment conducive to fetal growth, nutrient exchange, and immune tolerance. By the end of the third month, the capsular decidua fuses with the parietal decidua, obliterating the uterine cavity and ensuring sufficient space for the growing fetus and placenta. This process is driven by hormonal signals, including progesterone and relaxin, which stimulate significant cellular hyperplasia and hypertrophy in the uterus. Progesterone and relaxin are key hormones driving uterine preparation for pregnancy. Progesterone stimulates endometrial stromal cell proliferation and differentiation into decidual cells, essential for implantation and fetal tolerance. Relaxin remodels the uterine extracellular matrix, promotes angiogenesis, and relaxes uterine musculature to accommodate growth. Together, these hormones ensure the structural and functional changes required for a successful pregnancy. Supporting references include Stute et al. [27] on progesterone’s role in endometrial transformation and Sherwood et al. [28] on relaxin’s contributions to uterine remodeling. Additionally, molecular factors such as VEGF and insulin-like growth factor (IGF) promote vascular and tissue expansion, underscoring the dynamic interplay between maternal and placental adaptations during early pregnancy [29].

### 2.1. Early Placental Development

The placenta is composed of two distinct portions: the fetal portion, represented by the chorion (including the chorionic plate and chorionic villi), and the maternal portion, derived from the basal decidua. The formation of the utero-placental circulatory system begins as early as day 9 post-fertilization, with the appearance of vascular spaces within the rapidly expanding syncytiotrophoblast layer, referred to as trophoblastic lacunae [30]. These lacunae coalesce to form an interconnected network of sinusoidal spaces, which are essential for initiating maternal-fetal exchange [31].

As invasive trophoblast cells penetrate the maternal endometrial blood vessels, the trophoblastic lacunae fill with maternal blood, establishing the primitive utero-placental circulation. This process marks the onset of the hemochorial placentation unique to humans, characterized by the direct contact of maternal blood with fetal tissues. Maternal sinusoids, which originate from capillaries opening into the trophoblastic lacunae, undergo significant remodeling to support this system. The differential pressures between maternal arterial and venous vessels direct the flow of blood, ensuring efficient exchange of oxygen and nutrients [32].

The processes governing early placental development are tightly regulated by a network of molecular signals. VEGF and placental growth factor (PlGF) are critical for angiogenesis, promoting the formation and expansion of trophoblastic lacunae and maternal sinusoids. These growth factors act through the VEGFR-1 and VEGFR-2 receptors, ensuring the proper vascularization of the placental-uterine interface [33,34]. Concurrently, transforming TGF-β and MMPs facilitate trophoblast invasion by remodeling the extracellular matrix and degrading structural barriers in maternal tissues. The balance of pro-angiogenic and anti-angiogenic factors during this stage is essential for establishing the hemochorial placentation unique to humans [35]. Disruptions in these signaling pathways can impair utero-placental circulation, contributing to pregnancy complications such as preeclampsia or fetal growth restriction [36].

Concurrent with the formation of lacunae, the development of chorionic villi begins, marking a critical milestone in early placental development. These villi, which serve as the functional units of the placenta, facilitate maternal-fetal exchange and hormone production. Their development occurs in distinct stages [37]. Primary villi form by the end of the second week when cytotrophoblast cells proliferate and extend as short columns into the syncytiotrophoblast. By the third week, these structures transition into secondary villi as extraembryonic mesodermal cells infiltrate the cytotrophoblastic cores, establishing a mesodermal framework surrounded by syncytiotrophoblast. The final stage, tertiary villi, emerges at the end of the third week with the formation of a vascular network within the mesodermal core [38].

Functionally, tertiary villi are critical for initiating maternal-fetal exchange by connecting with the developing fetal vasculature in the chorionic plate and the connecting stalk, completing the feto-placental circulatory system. However, it is important to note that tertiary villi are not fully mature at this stage. Studies, such as those by Vogel and Turowski [39], indicate that these villi continue to branch and vascularize, with a small number of mature villi observed by gestational week 24. This branching increases the placental surface area, optimizing its efficiency in nutrient and gas exchange. Furthermore, the vascularization of tertiary villi enables the production of hormones such as human chorionic gonadotropin (hCG) and placental lactogen, which are essential for maintaining pregnancy and regulating fetal growth [12].

As the villous structures develop, several key cavities and membranes essential for placental and embryonic development also take shape. The amniotic cavity emerges as a small space within the epiblast layer of the bilaminar germ disc, forming around day 8–9 post-fertilization. The cells lining the roof of this cavity, known as amnioblasts, remain continuous with the cytotrophoblast shell, contributing to the structural stability of the developing embryo. Concurrently, hypoblast cells form the lining of the primitive yolk sac, which appears between days 10 and 12. This primitive yolk sac is later replaced by the smaller, definitive yolk sac by the end of the fourth week of gestation. The yolk sac not only provides early nutrient exchange but also plays a transient role in hematopoiesis, contributing to the formation of blood cells before the placenta assumes this function [40,41].

The formation of these structures is regulated by molecular signals such as VEGF and fibroblast growth factor-2 (FGF-2), which promote the proliferation and differentiation of mesodermal cells derived from the primitive yolk sac. These mesodermal cells contribute to the formation of the extraembryonic mesoderm, a critical tissue layer that supports placental structure. Cavities within the extraembryonic mesoderm coalesce to create the chorionic cavity (extraembryonic coelom) by days 12–15. VEGF also facilitates the vascularization of the yolk sac, ensuring its role in nutrient delivery during this phase [42]. As development progresses through the third and fourth weeks, the amniotic sac expands to fill the chorionic cavity, bringing the mesodermal tissues of the amnion and chorion into close apposition. By the end of the fourth week, the amnion and chorion remain in apposition yet separable, a feature that allows for structural flexibility and adaptability throughout gestation [26].

The development of the umbilical cord, which serves as the lifeline between the fetus and the placenta, is an important component of placental embryology. Around the fifth week of gestation, several embryonic structures become incorporated into the connecting stalk, which will ultimately form the umbilical cord. These structures include the yolk sac stalk with its vitelline vessels, the allantois, and the umbilical vessels, which are essential for nutrient and gas exchange between the fetus and the placenta. At this stage, intestinal loops temporarily protrude into the connecting stalk through the omphalomesenteric duct, a process referred to as physiological herniation. By the end of the embryonic period, typically around the 10th week of gestation, the intestines return to the abdominal cavity, and the yolk sac is obliterated [43].

In normal development, the retraction of intestinal loops is completed by the 12th week of gestation, accompanied by proper closure of the abdominal wall. In contrast, an omphalocele occurs when the intestines fail to return to the abdominal cavity, remaining outside the body and covered by a membranous sac. This anomaly results from disrupted embryological timing or incomplete closure of the lateral folds, underscoring the importance of precise coordination during umbilical cord and abdominal wall development [44].

### 2.2. Development of the Villous Tree

The chorionic plate, which forms the fetal surface of the placenta, is a fundamental structure in placental development. Covered by the amnion, it contains the fetal blood vessels that branch from the umbilical cord to supply the villous tree. These villous trees extend into the intervillous space, establishing a network for efficient maternal-fetal exchange. Throughout gestation, the villous tree undergoes a process of branching morphogenesis, forming new villous subtypes that expand the surface area for exchange. In contrast, maturation of the villous tree involves structural refinement of existing villi to optimize their functional capabilities [45].

**Figure 1:**
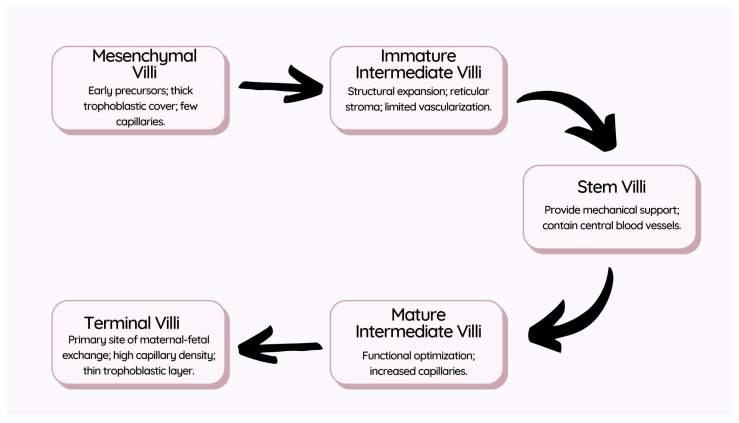
Development and Maturation of the Villous Tree.

The development and maturation of the villous tree can be categorized into five distinct villous types: mesenchymal villi, immature intermediate villi, stem villi, mature intermediate villi, and terminal villi (Figure 1). Each subtype exhibits unique structural characteristics tailored to its specific functional role. For instance, mesenchymal villi, which predominate in early pregnancy, serve as precursors for later villous subtypes. Terminal villi, which form primarily in the third trimester, are specialized for nutrient and gas exchange. The transition from development to maturation involves extensive differentiation and vascularization, ensuring that the placenta meets the increasing metabolic demands of the growing fetus. This differentiation process is distinct from the remodeling of maternal spiral arteries, which supports placental perfusion by establishing low-resistance blood flow [46].

Villous development and maturation are driven by dynamic molecular signaling processes that regulate trophoblast behavior, angiogenesis, and vascular remodeling. Trophoblast differentiation into cytotrophoblast and syncytiotrophoblast layers during villous formation is modulated by pathways such as Wnt/β-catenin and BMP (Bone Morphogenetic Protein), which influence cellular proliferation and polarity [47]. During villous maturation, epigenetic modifications, including DNA methylation and histone acetylation, play critical roles in fine-tuning gene expression for structural and functional specialization [48].

Further refinement of the vascular network within tertiary and terminal villi is regulated by endothelial nitric oxide synthase (eNOS), which promotes vasodilation and increases blood flow to meet the growing fetal demands. Additionally, angiopoietins (Ang-1 and Ang-2) coordinate with Tie receptors to stabilize nascent blood vessels and ensure vascular integrity [49]. In terminal villi, hypoxic conditions during early pregnancy can induce a switch in signaling cascades, activating transcription factors such as HIF-1α. This response facilitates oxygen transport and promotes villous branching under such conditions. However, oxygen levels vary across gestational stages, and hypoxia-related effects are more prominent during the early phases of placental development [50]. These regulatory mechanisms are essential for creating a villous tree capable of adapting to the evolving metabolic needs of the fetus and maintaining efficient maternal-fetal exchange [51].

#### 2.2.1. Mesenchymal Villi

Mesenchymal villi are the first generation of tertiary villi, appearing around the fifth week of gestation and serving as precursors for all other villous types. These villi are characterized by a thick trophoblastic cover with prominent cytotrophoblasts and the highest mitotic index among all villous types, reflecting their role in early placental growth. The stromal core is primitive, composed of loosely arranged collagen, fibroblasts, and a few Hofbauer cells, with poorly developed fetal capillaries. In the early stages of gestation, mesenchymal villi are the primary site for villous proliferation, maternal-fetal exchange, and endocrine activity. However, their prominence diminishes as pregnancy progresses. Studies indicate that mesenchymal villi largely disappear by gestational week 20, transitioning into more specialized villous subtypes such as mature intermediate and terminal villi. By term, mesenchymal villi comprise less than 1% of the placental volume and are primarily restricted to small areas on the surfaces of immature intermediate villi within the central regions of the villous trees [38].

#### 2.2.2. Immature Intermediate Villi

Immature intermediate villi emerge around the eighth week of gestation and dominate the villous population from the 14th to the 20th week. These villi develop through the maturation of mesenchymal villi during the first two trimesters and are critical for the structural expansion of the villous tree. Characterized by their bulbous shape, thick trophoblastic cover, and prominent cytotrophoblast layer, immature intermediate villi also exhibit a distinctive reticular stroma containing fluid-filled stromal channels. The stroma harbors Hofbauer cells, which play a role in immune regulation, while fetal capillaries remain underdeveloped during this stage. Beginning at approximately the eighth week, immature intermediate villi serve as growth centers for the villous trees, facilitating the formation of new mesenchymal villi. As gestation progresses, these villi undergo structural transitions, eventually giving rise to stem villi, which provide mechanical stability to the villous tree. By term, only rare clusters of immature intermediate villi persist, primarily located in the central regions of the villous trees. These clusters account for less than 5% of the placental volume at term, reflecting their gradual transformation into more specialized villous subtypes [52].

#### 2.2.3. Stem Villi

Stem villi begin to appear around the eighth week of gestation, emerging through the gradual transformation of immature intermediate villi. These villi form the large “trunks” and “branches” of the villous trees, as well as the anchoring villi, which secure the placenta to the maternal decidua. The transition from immature intermediate villi to stem villi is marked by distinct morphological changes, including the development of a well-defined media and adventitia in the blood vessels and an increase in connective tissue density. Histologically, stem villi are characterized by a thick trophoblastic cover, with cytotrophoblasts remaining visible on approximately 20% of the villous surfaces. The stroma of stem villi is highly condensed, containing bundles of collagen fibers, scattered fibroblasts, and rare immune cells, such as macrophages and mast cells. Larger stem villi contain a central artery and corresponding vein, along with smaller arterioles, venules, and superficial paravascular capillaries. Their primary role is to provide mechanical support to the placental structure, with minimal direct involvement in maternofetal exchange [53].

#### 2.2.4. Mature Intermediate Villi

Mature intermediate villi begin to develop around gestational week 16, emerging from mesenchymal villi as part of the transition to a more vascularized and specialized villous tree. These villi are typically long and slender, often forming a zigzag configuration, with their surfaces supporting the development of terminal villi. The stroma of mature intermediate villi consists of unoriented, loose bundles of connective tissue fibers interspersed with numerous capillaries, small terminal arterioles, and collecting venules. Cross-sections of these villi contain less than 50% vascular lumens, reflecting their specialized role in feto-maternal exchange by facilitating efficient nutrient and gas transfer. By gestational week 16, mature intermediate villi constitute approximately 30% of the placental villous population, and they remain prominent throughout pregnancy, comprising approximately 30% of the placental volume at term [54].

#### 2.2.5. Terminal Villi

Terminal villi represent the final and most specialized structures of the villous tree, forming as grape-like outgrowths from mature intermediate villi during the third trimester. These villi are uniquely adapted for feto-maternal exchange, with their structure characterized by thin trophoblastic coverings closely apposed to sinusoidally dilated capillaries. The intimate contact between fetal and maternal blood across the vasculosyncytial membranes facilitates efficient nutrient and gas transfer. Unlike other villous types, terminal villi are defined by their vascular architecture, with vascular lumens comprising at least 50% of the stromal volume and containing only capillaries and sinusoids. Their formation is largely driven by passive capillary growth and coiling, which stretch the trophoblast and thin the membranes for optimal exchange. At term, terminal villi dominate the placental villous architecture, comprising approximately 45% of the placental volume and 60% of cross-sectional areas, underscoring their critical role in sustaining fetal growth during late gestation [55].

The development of the villous tree is a sequential process, with each villous type evolving to fulfill specific structural and functional roles within the placenta. As shown in Table 2, the progression from mesenchymal villi to terminal villi reflects increasing specialization, from early stages focused on growth and proliferation to mature forms optimized for efficient maternal-fetal exchange. Each villous type contributes uniquely to the placental structure, supporting both mechanical stability and the exchange capacity essential for fetal development.

## 3. Placental-Uterine Interface and Circulatory Systems

The placental-uterine interface is a critical and dynamic zone where maternal and fetal tissues interact to support the developing fetus. This interface is essential for the exchange of nutrients, gases, and waste products, which sustains fetal growth and health throughout pregnancy. Beyond its role in material exchange, the placental-uterine interface also plays a vital immunological function, helping the maternal immune system tolerate the presence of fetal cells, which carry paternal antigens. This tolerance is fundamental to preventing immune rejection and maintaining pregnancy stability [59].

The primary components of this interface include the decidua, spiral arteries, and chorionic villi. The decidua, derived from the maternal endometrium, undergoes specific changes to support trophoblast invasion and establish a receptive environment for the placenta. The spiral arteries, which supply blood to the placenta, are remodeled by trophoblast cells to increase blood flow and create a low-resistance circulatory system essential for fetal oxygenation. Meanwhile, the chorionic villi, projections from the fetal side, extend into the intervillous space and act as the primary site of maternal-fetal exchange. These components together create a complex and highly regulated interface, ensuring that the needs of the growing fetus are met while also maintaining maternal health and pregnancy viability [60].

### 3.1. Structural Components of the Placental-Uterine Interface

The placental-uterine interface is composed of several distinct but interconnected structural components, each with unique characteristics and functions that contribute to a supportive environment for fetal development. Understanding these components is essential to appreciating how the maternal and fetal systems interact seamlessly to sustain pregnancy [61].

#### 3.1.1. Decidua

The decidua is the specialized endometrial tissue of the uterus that undergoes profound changes during pregnancy. It is divided into three regions: the decidua basalis, decidua parietalis, and decidua capsularis, each playing a specific role in the establishment and maintenance of the placenta.

- Decidua Basalis: This is the portion of the endometrium located directly beneath the implanted embryo and is in direct contact with the chorionic villi. The decidua basalis undergoes extensive remodeling, supporting the attachment and invasion of trophoblast cells from the placenta. It is rich in blood vessels and maternal immune cells, which play a role in both nutrient supply and immunological adaptation, helping prevent fetal rejection.
- Decidua Parietalis: This region lines the remaining uterine cavity, not directly adjacent to the implantation site. It undergoes mild changes during pregnancy but does not directly interact with the chorionic villi. However, the decidua parietalis contributes to the structural integrity of the uterine wall and, in later stages, fuses with the decidua capsularis as the amniotic sac expands to fill the uterine cavity.
- Decidua Capsularis: This portion initially covers the embryo and separates it from the uterine cavity. As pregnancy progresses and the fetus grows, the decidua capsularis stretches and thins. By around the second trimester, it typically fuses with the decidua parietalis, leading to the obliteration of the uterine cavity. This fusion provides additional support to the expanding gestational sac and maintains the structural cohesion of the placental environment [62].

**Figure 2:**
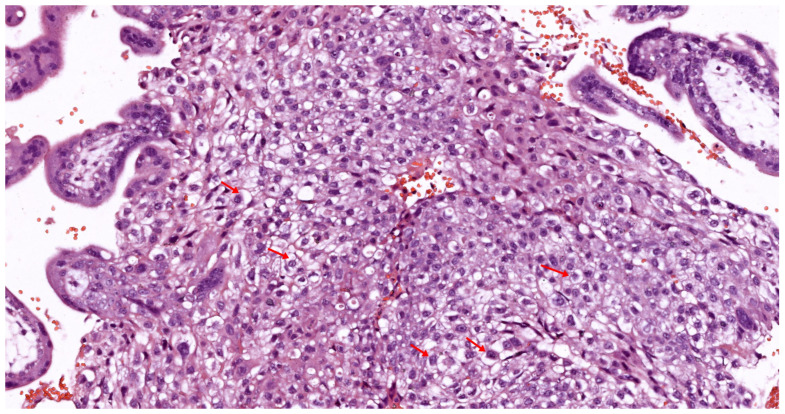
Histological section of the decidua at 20× magnification, showing large, polygonal decidual cells (indicated by red arrows) with abundant cytoplasm rich in glycogen and lipids.

Histologically, the decidua is characterized by large, polygonal decidual cells that have differentiated from endometrial stromal cells under the influence of progesterone. These decidual cells are rich in glycogen and lipids, providing a source of energy and nutrients to support early trophoblast invasion. As shown in Figure 2, the decidua is composed of specialized decidual cells that create an optimal environment for implantation and early placental growth. Additionally, the decidual cells play a critical role in modulating the immune environment by producing cytokines and growth factors that favor tolerance toward fetal cells. This transformation, known as decidualization, creates an optimal environment for implantation and placental growth, marking the decidua as a critical player in the placental-uterine interface [63].

#### 3.1.2. Trophoblast Invasion and Decidual Reaction

**Figure 3:**
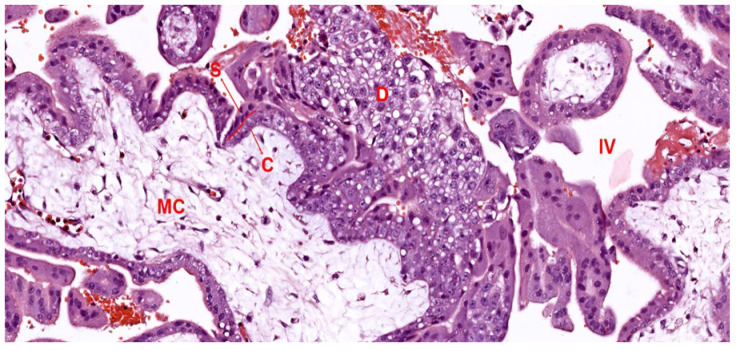
Histological section of the placenta at 20× magnification, illustrating the the structure of chorionic tertiary villi within the intervillous space. The image highlights various components essential for placental function, including the mesenchymal core (MC), cytotrophoblast cells (C), syncytiotrophoblast cells (S), decidual cells (D), and intervillous space (IV).

The process of trophoblast invasion initiates the formation of a stable placental-uterine connection, vital for the efficient delivery of maternal blood to the developing placenta. Derived from the outer blastocyst layer, trophoblast cells diversify into two specialized types—cytotrophoblasts and syncytiotrophoblasts—each playing a unique role in the architecture and functionality of the placenta (Figure 3) [64].

- Cytotrophoblasts: These are characterized by their single nucleus, comprise the inner trophoblast layer, and exhibit prolific growth. During placental maturation, these cells differentiate further into villous cytotrophoblasts—supporting the villous structure—and extravillous cytotrophoblasts, which actively penetrate maternal tissues to remodel spiral arteries and secure the placental position. This invasion is crucial as it allows the cytotrophoblasts to replace the endothelial lining of maternal blood vessels, creating a low-resistance pathway that facilitates increased blood flow to the placenta [65].
- Syncytiotrophoblasts: Formed by the fusion of cytotrophoblast cells, the syncytiotrophoblast layer represents the outermost barrier of the placenta, in direct contact with maternal blood. This multinucleated layer plays an essential role in nutrient and gas exchange, hormone production, and immunological protection. By secreting hCG, the syncytiotrophoblast sustains the corpus luteum in the early stages of pregnancy, ensuring continued progesterone production until the placenta can take over hormone synthesis. Furthermore, the syncytiotrophoblasts express specific proteins that help evade maternal immune detection, supporting the immune tolerance necessary for a successful pregnancy [66].

The interaction between the invading trophoblast cells and the maternal decidua is a coordinated process involving both mechanical and biochemical signaling. As cytotrophoblasts invade the maternal tissue, they secrete enzymes that degrade the extracellular matrix, facilitating deeper penetration into the decidua basalis. This invasion is accompanied by a decidual reaction, where decidual cells respond by producing various cytokines and growth factors that regulate trophoblast behavior and modulate local immune responses. The immune cells in the decidua, including macrophages and uNK cells, play supportive roles in trophoblast invasion, promoting vascular remodeling while maintaining an environment that is permissive yet controlled [67].

Overall, the decidua and trophoblast populations form an intricate, highly regulated interface that balances the requirements for nutrient delivery, immune tolerance, and structural integrity. The successful establishment of this interface is foundational to the development of the placenta and the maintenance of a healthy pregnancy [68].

### 3.2. Molecular Regulation of Placental Development

The placenta is a dynamic organ whose development relies on a cascade of molecular events orchestrating trophoblast differentiation, vascular remodeling, and immune modulation. These pathways ensure the establishment of an efficient maternal-fetal interface while adapting to the evolving metabolic demands of the growing fetus [13].

Trophoblast differentiation is initiated upon implantation, with cytotrophoblasts differentiating into two key populations: syncytiotrophoblasts and extravillous trophoblasts (EVTs). Wnt/β-catenin and BMP (Bone Morphogenetic Protein) pathways regulate early trophoblast lineage commitment by promoting cellular proliferation and polarity. The expression of Notch signaling components, such as Notch1 and Delta-like ligand 4 (DLL4), further modulates trophoblast invasion and syncytialization [69].

The syncytiotrophoblast layer, responsible for hormonal secretion and nutrient transfer, employs molecular regulators such as Syncytin-1 and GCM1 (glial cells missing-1) to maintain its multinucleated architecture. Meanwhile, EVTs utilize integrins (ITGα5β1) and matrix metalloproteinases (e.g., MMP-2 and MMP-9) to invade maternal tissues and remodel the spiral arteries. Dysregulation of these pathways has been linked to shallow invasion and complications such as preeclampsia [70].

Vascularization within the developing villous tree is regulated by a balance between pro-angiogenic and anti-angiogenic factors. VEGF (vascular endothelial growth factor) and PlGF (placental growth factor) act through their receptors VEGFR-1 and VEGFR-2, stimulating endothelial cell proliferation and vessel formation. In parallel, angiopoietins (Ang-1 and Ang-2) interact with Tie2 receptors, stabilizing nascent vessels while allowing for adaptive remodeling [71].

Low oxygen tension in the early placenta drives the expression of HIF-1α (hypoxia-inducible factor-1α), which upregulates VEGF and promotes vascular network expansion. However, as the pregnancy progresses, increased oxygen availability induces a shift in signaling pathways, promoting vascular stabilization. Endothelial nitric oxide synthase (eNOS) contributes to vasodilation and ensures sufficient blood flow to meet fetal demands [72].

Epigenetic regulation is crucial for the fine-tuning of gene expression during villous maturation. DNA methylation patterns and histone modifications (e.g., acetylation via histone acetyltransferases) regulate the differentiation of cytotrophoblasts and the structural remodeling of villi. Specific microRNAs, such as miR-210 and miR-34a, modulate HIF-1α activity and trophoblast invasion, ensuring appropriate villous development. The integration of these epigenetic mechanisms supports the transition of villi into their specialized subtypes, such as terminal villi, where efficient nutrient and gas exchange occurs [73].

The placenta establishes a finely tuned immune microenvironment to avoid fetal rejection. Extravillous trophoblasts express HLA-G, a non-classical MHC molecule that suppresses maternal immune activation. Decidual natural killer (dNK) cells, the predominant immune cells in the decidua, interact with trophoblasts via KIR (killer-cell immunoglobulin-like receptor) signaling, facilitating arterial remodeling. Additionally, macrophages and regulatory T cells (Tregs) secrete anti-inflammatory cytokines such as IL-10 and TGF-β, maintaining immune tolerance. Aberrant immune modulation is associated with recurrent pregnancy loss and complications such as preeclampsia [74].

The syncytiotrophoblast layer acts as an endocrine hub, producing key hormones essential for pregnancy maintenance. Human chorionic gonadotropin (hCG), regulated by transcription factors such as CREB (cyclic AMP response element-binding protein), sustains corpus luteum function during early pregnancy. Placental lactogen (hPL) and progesterone are critical for modulating maternal metabolism and preparing for fetal growth.

### 3.3. Establishment and Regulation of Uteroplacental Circulation

**Figure 4:**
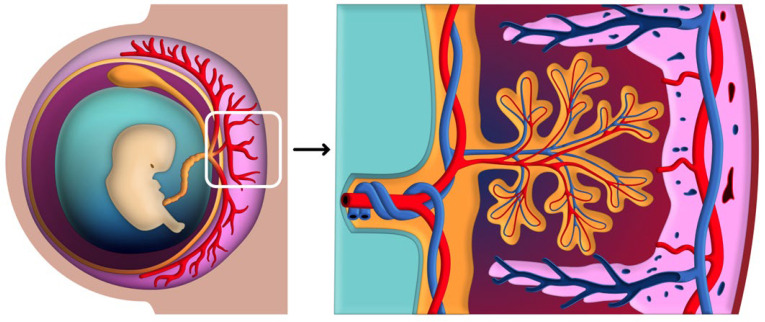
Illustration of the developing placenta and early uteroplacental circulation. The left side shows an embryo within the amniotic cavity surrounded by the trophoblast and early placental structures. The right side zooms into the placental interface, highlighting the chorionic villi’s branching structure within the intervillous space, where maternal blood circulates to facilitate nutrient and gas exchange.

The establishment of the uteroplacental circulation is essential for meeting the metabolic needs of the developing fetus. This system is created through a combination of trophoblast invasion, spiral artery remodeling, and the formation of the intervillous space, all of which work together to ensure efficient blood flow and nutrient delivery to the placenta. In addition, hormonal and biochemical signals dynamically regulate blood flow to meet the fetus’s increasing demands as pregnancy progresses. Figure 4 illustrates the early stages of placental development and the establishment of the uteroplacental circulation. This schematic representation emphasizes the structural complexity of the chorionic villi within the intervillous space, which is essential for efficient maternal-fetal exchange [75].

#### 3.3.1. Invasion and Remodeling of Spiral Arteries

A defining feature of uteroplacental circulation is the intricate interplay between villous structures and maternal blood flow. The transformation of maternal spiral arteries is critical to this process, ensuring an adequate supply of oxygen and nutrients to the developing fetus. Early in pregnancy, spiral arteries undergo extensive remodeling, orchestrated by invasive extravillous trophoblasts. This process begins with cytotrophoblasts infiltrating the decidua, where they differentiate into invasive extravillous trophoblasts. These cells penetrate the walls of the spiral arteries, replacing the endothelial lining and partially disrupting the smooth muscle layers. This remodeling expands the arterial lumen, converting the high-resistance vessels into low-resistance conduits capable of delivering large volumes of blood to the intervillous space [76].

Within the intervillous space, maternal blood bathes the floating villi, which play a pivotal role in maternal-fetal exchange. The dense network of capillaries in terminal villi, coupled with the thin vasculosyncytial membranes, facilitates efficient diffusion of gases, nutrients, and waste products. The unique branching architecture of the villous tree, with terminal villi extending into the intervillous space, ensures maximal contact with maternal blood flow. This structural adaptation, combined with the vascular remodeling of the spiral arteries, optimizes the perfusion of maternal blood through the placenta, sustaining the metabolic demands of the growing fetus [77].

The significance of this remodeling lies in the resulting shift in blood flow characteristics. Normally, spiral arteries are narrow, high-pressure vessels that restrict blood flow. However, when remodeled, these vessels deliver a high volume of blood directly into the intervillous space without significant pulsatility, providing a constant, low-resistance blood supply that is essential for adequate placental perfusion. This transformation ensures a stable and continuous flow of oxygen and nutrients to the placenta, meeting the needs of the growing fetus [78].

Studies from the placenta and extraembryonic membranes provide insights into the physiological mechanisms behind this process. Research has shown that the extent and timing of spiral artery remodeling are crucial for placental function and pregnancy success. Insufficient remodeling can lead to complications such as preeclampsia and fetal growth restriction, as the high-resistance arteries fail to deliver adequate blood flow to the intervillous spaces. The role of trophoblasts in transforming these vessels into efficient conduits highlights the adaptive complexity of the placental-uterine interface, which evolves to sustain fetal development [79].

#### 3.3.2. Intervillous Space and Maternal-Fetal Blood Flow

The intervillous space is the central region of the placenta where maternal blood bathes the chorionic villi, enabling direct exchange between maternal and fetal circulations. Blood flows into the intervillous space through the remodeled spiral arteries, filling the area around the chorionic villi, which contain the fetal capillary networks. This arrangement is particularly effective in the human placenta, which exhibits hemochorial placentation—a type where maternal blood is in direct contact with the syncytiotrophoblast layer of the chorionic villi [25].

In hemochorial placentation, the syncytiotrophoblast, a continuous outer layer of the chorionic villi, serves as the primary barrier between maternal blood and fetal tissue. This thin layer allows for rapid diffusion of gases and nutrients from maternal blood to fetal circulation while also facilitating the removal of fetal waste products. The positioning of fetal capillaries close to the syncytiotrophoblast surface minimizes the distance for diffusion, enhancing the efficiency of exchange. The intervillous space thus acts as an intermediary environment, balancing maternal blood flow with the delicate demands of the fetal circulation. This structure also provides a protective mechanism. While allowing for efficient exchange, the intervillous space limits direct cellular contact between maternal and fetal cells, thus reducing immunological risks to the fetus. The complex arrangement and management of blood flow within the intervillous space are hallmarks of placental specialization, optimized for supporting the fetus in a way that protects both fetal and maternal health [80].

#### 3.3.3. Hormonal and Biochemical Factors Influencing Blood Flow

The regulation of uteroplacental blood flow involves a dynamic interplay of hormones and biochemical signals, which adapt circulation to the growing needs of the fetus. Progesterone and human chorionic gonadotropin (hCG) are among the key hormones involved in maintaining optimal blood flow to the placenta. Progesterone, which is initially produced by the corpus luteum and later by the placenta itself, helps maintain uterine quiescence and enhances vascular remodeling in the early stages of pregnancy. It also modulates the local immune environment, promoting tolerance of the fetal cells invading the uterine tissue. Human chorionic gonadotropin (hCG), secreted by the syncytiotrophoblast, plays multiple roles in early placental development, including promoting the survival and function of the corpus luteum. Additionally, hCG may influence local vascular growth by encouraging trophoblast invasion and angiogenesis, which are essential for establishing an effective placental blood supply. Elevated hCG levels in early pregnancy help maintain the hormonal environment necessary for sustaining uterine and placental functions [81].

Biochemical signals such as nitric oxide (NO), prostaglandins, and various cytokines also contribute to vascular regulation within the uteroplacental unit. Nitric oxide, in particular, is a vasodilator that helps relax the smooth muscles of blood vessels, reducing vascular resistance and promoting blood flow through the remodeled spiral arteries. Local synthesis of NO in the placenta and surrounding uterine tissues ensures that blood flow remains adequate as the demands of the fetus increase. Prostaglandins, which are lipid compounds involved in inflammation and vascular remodeling, further support placental vascular adaptation by promoting vasodilation and regulating blood vessel permeability. In response to the fetus’s increasing metabolic demands, the uteroplacental circulation exhibits remarkable adaptability. Blood vessel diameter can increase, and the intervillous space can expand to accommodate the growing volume of maternal blood required to meet fetal needs. This adaptability ensures that the placental blood supply is continually optimized to support fetal growth throughout gestation [82,83].

In conclusion, the establishment and regulation of uteroplacental circulation involve a combination of structural changes, hormonal influence, and biochemical signals. This system ensures that the placenta can provide a steady, low-resistance flow of maternal blood to support fetal development, adapting dynamically as pregnancy progresses. Through the coordinated action of trophoblasts, hormonal regulators, and biochemical factors, the uteroplacental circulation maintains the delicate balance required for a healthy pregnancy.

### 3.4. Immunological Aspects of the Placental-Uterine Interface

The placental-uterine interface presents a unique immunological environment, where the maternal immune system must recognize and tolerate the semi-allogeneic fetus. This tolerance is essential to prevent immune-mediated rejection while maintaining immune defenses to protect against infections. The placenta actively contributes to establishing maternal immune tolerance through various mechanisms, including specialized trophoblast behavior and interaction with maternal immune cells within the decidua [84].

To maintain a successful pregnancy, the placenta creates a local immunological environment that prevents maternal immune cells from targeting fetal cells, which express paternal antigens. This immune tolerance is facilitated by both physical and molecular adaptations. The syncytiotrophoblast layer, which forms the outer barrier of the chorionic villi, is a fused, multinucleated cell layer that lacks the classical human leukocyte antigen (HLA) molecules. By not expressing HLA class I and II antigens, the syncytiotrophoblast minimizes the activation of maternal immune cells against the fetal tissue [85].

Extravillous trophoblasts (EVTs) have a central role in immune tolerance by expressing specific non-classical HLA molecules, particularly HLA-G. This HLA variant is crucial for immune modulation, as it does not provoke an aggressive maternal immune response and instead fosters tolerance. HLA-G on extravillous trophoblasts interacts with receptors on maternal immune cells, such as uNK cells, promoting an immune-protective environment that supports trophoblast invasion and placental attachment. HLA-G expression is considered a key adaptation that allows EVTs to invade the maternal decidua and remodel spiral arteries without triggering an adverse immune reaction [86].

The decidua contains a diverse array of maternal immune cells that are essential for maintaining a balanced immune response at the maternal-fetal interface. Among these, decidual macrophages, uNK cells, and T-regulatory (Treg) cells have an essential role. Decidual macrophages contribute to tissue remodeling and clear cellular debris, supporting a clean and adaptive environment for placental development. uNK cells, which are abundant in the decidua, interact with HLA-G on trophoblasts, secreting cytokines and growth factors that promote trophoblast invasion and vascular remodeling. Unlike peripheral NK cells, uNK cells are more focused on promoting placental development than inducing cytotoxic responses [87].

Tregs in the decidua also contribute to immune tolerance by suppressing maternal immune responses that might target fetal antigens. Through cytokine production and cell-to-cell interactions, Tregs help modulate the activity of other immune cells, fostering a tolerant environment necessary for fetal survival. These immune cells work in concert, creating an immunologically permissive environment that supports the invasive and nutrient-exchanging functions of the placenta [88].

In summary, the immunological adaptations at the placental-uterine interface reflect a finely tuned balance between tolerance and defense, orchestrated through specialized trophoblast functions and the cooperative actions of maternal immune cells. This unique immune environment is essential for a successful pregnancy, allowing fetal development to proceed uninterrupted within the maternal host.

### 3.5. Pathophysiological Implications and Clinical Relevance

When these processes are disrupted, the placenta cannot adequately support the needs of the developing fetus, leading to a range of pregnancy complications with both immediate and long-term health impacts [89].

#### 3.5.1. Complications Arising from Defective Trophoblast Invasion

Insufficient trophoblast invasion and inadequate remodeling of the spiral arteries are associated with conditions such as preeclampsia and intrauterine growth restriction (IUGR). In a healthy pregnancy, extravillous trophoblasts invade the maternal decidua and modify the spiral arteries, transforming them from high-resistance vessels into low-resistance conduits capable of supplying increased blood flow to the placenta. However, when trophoblast invasion is incomplete or spiral artery remodeling is suboptimal, the uteroplacental blood flow is restricted. This restricted blood flow can lead to placental hypoxia, setting off a cascade of pathological responses [90].

Preeclampsia, a condition characterized by hypertension and proteinuria in the mother, is often linked to inadequate spiral artery remodeling. The resultant placental hypoxia induces the release of antiangiogenic factors into the maternal circulation, leading to widespread endothelial dysfunction and the clinical symptoms of preeclampsia. Similarly, insufficient blood flow to the placenta due to poor spiral artery remodeling can limit fetal nutrient and oxygen supply, resulting in IUGR. In these cases, the fetus’s growth potential is compromised, increasing the risk of neonatal complications and adverse outcomes [91].

#### 3.5.2. Impact on Long-Term Health

The consequences of abnormal placental development extend beyond the immediate pregnancy and birth outcomes, affecting both maternal and child health long-term. Women who experience preeclampsia are at an elevated risk for cardiovascular diseases, including hypertension and stroke, later in life. For the child, being born with IUGR is associated with increased susceptibility to metabolic disorders, such as type 2 diabetes and obesity, as well as cardiovascular disease in adulthood. This phenomenon, known as “fetal programming”, suggests that adverse conditions in utero can alter physiological and metabolic pathways, predisposing individuals to chronic health conditions [92].

In conclusion, defects in placental development, particularly in trophoblast invasion and spiral artery remodeling, can have severe pathophysiological consequences. These placental abnormalities not only impact immediate pregnancy outcomes but also have significant implications for the lifelong health of both mother and child, highlighting the importance of early detection and intervention in high-risk pregnancies.

## 4. Morphology and Functional Layers of the Placenta

The placenta is composed of distinct layers, each with specialized functions that support maternal-fetal exchange, structural stability, and immunological defense. These layers include the chorionic plate, basal plate, and the intervillous space. Each layer contributes to the intricate architecture of the placenta, facilitating its role as a highly adaptive and functional interface between mother and fetus [42].

### 4.1. Chorionic Plate

The chorionic plate forms the fetal side of the placenta and is the surface through which umbilical vessels pass from the fetus to the placenta. Structurally, the chorionic plate is a connective tissue layer covered by the amnion and lined with trophoblast cells. This layer not only provides the anchoring surface for the villous tree but also ensures that maternal and fetal blood supplies remain separate, except at the sites of exchange within the villi. Within the chorionic plate, large fetal blood vessels branch out from the umbilical cord and further divide to supply blood to the villous tree. These vessels are crucial for transporting oxygenated blood from the placenta to the fetus and returning deoxygenated blood to the maternal circulation. The chorionic plate’s robust connective tissue provides support and maintains the structural integrity of these vessels, preventing compression as they branch into smaller vessels within the villi. Histologically, the chorionic plate is characterized by a dense arrangement of connective tissue fibers, particularly collagen, providing tensile strength that helps sustain the mechanical demands of pregnancy [93].

While the chorionic plate supports the fetal vascular network and serves as the site of attachment for the villous tree, the basal plate on the maternal side plays a crucial role in anchoring the placenta, ensuring stable positioning, and facilitating maternal-fetal exchange.

### 4.2. Basal Plate

The basal plate constitutes the maternal side of the placenta and is directly attached to the uterine wall. This layer forms the foundation from which placental villi extend, embedding into the maternal decidua. Structurally, the basal plate consists of decidual cells, extravillous trophoblasts, Langhans fibrinoid, and Nitabuch fibrinoid, all organized to ensure effective maternal blood flow to the intervillous spaces. Langhans fibrinoid is a fibrin-like extracellular material deposited in the basal plate, contributing to the structural stability of the maternal-fetal interface. This layer plays a regulatory role in limiting excessive trophoblast invasion and maintaining the integrity of the placental architecture. In addition to structural support, Langhans fibrinoid may influence cellular signaling pathways between maternal and fetal tissues, ensuring controlled trophoblast activity throughout gestation. Nitabuch fibrinoid forms a specialized boundary layer between the trophoblast and the decidua basalis. This layer prevents excessive trophoblast invasion into the uterine tissue, maintaining a critical balance that safeguards maternal health while supporting fetal growth. Nitabuch fibrinoid is particularly important in preventing pathological conditions such as placenta accreta, where trophoblast cells invade too deeply into the myometrium [94,95].

**Figure 5:**
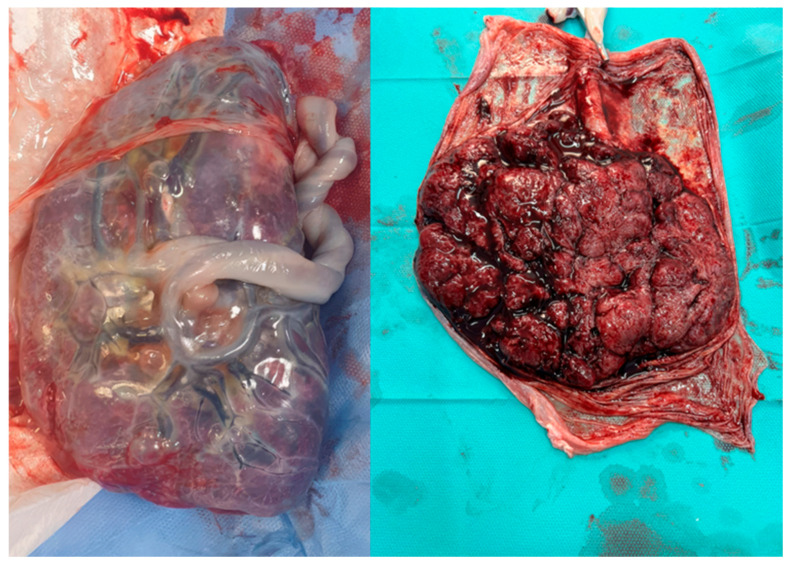
The left image shows a placenta with a magistral pattern of chorionic plate vessels originating from a marginal insertion of the umbilical cord. In this configuration, blood vessels radiate from the edge of the placenta, distributing blood to the chorionic villi. The right image displays the maternal floor of a term placenta, revealing numerous well-defined cotyledons or lobules. These cotyledons, which are the functional units of the basal plate, contain chorionic villi essential for nutrient and gas exchange between maternal and fetal blood within the intervillous space. The organized arrangement of these cotyledons enhances placental efficiency by maximizing the surface area available for maternal-fetal exchange. Macroscopy images, representative of placental anatomy, obtained during the authors’ clinical practice within the Gynecology ward (private collection).

The basal plate also contains an organized network of cotyledons (lobules), each acting as a functional unit that houses chorionic villi. These villi facilitate the exchange of nutrients and gases between maternal and fetal blood (Figure 5). The structured arrangement of cotyledons ensures efficient blood distribution across the maternal floor, meeting the growing demands of the fetus as gestation progresses. Additionally, extravillous trophoblasts within the basal plate invade maternal spiral arteries, remodeling them into low-resistance vessels to increase blood flow to the placenta. This process ensures a steady and adequate blood supply to the intervillous spaces, which is critical for the placenta’s role in fetal development. Thus, the basal plate serves as a vital layer for both structural attachment and functional exchange, ensuring stable positioning of the placenta and adequate blood flow. The inclusion of Langhans and Nitabuch fibrinoid layers highlights the intricate regulatory mechanisms at the maternal-fetal interface, which are essential for a healthy pregnancy [96].

### 4.3. Intervillous Space

The intervillous space is a central feature of the placenta, serving as the primary area where maternal blood directly bathes the fetal chorionic villi. This space is formed by the branching villous tree, which suspends numerous villi into the intervillous area, maximizing the surface area for nutrient and gas exchange. Maternal blood is supplied to the intervillous space via spiral arteries, which deliver oxygen-rich blood under low pressure, allowing it to pool around the villi and enabling efficient diffusion. The intervillous space is filled with maternal blood and maintained at a low pressure to prevent mechanical damage to the delicate villous structures. The close proximity of maternal blood to the fetal capillaries within the villi facilitates rapid exchange, with minimal diffusion distance for oxygen, nutrients, and waste products. The architecture of the intervillous space, along with the thin syncytiotrophoblast layer covering the villi, is particularly well-adapted to the metabolic demands of the fetus, allowing efficient gas exchange and nutrient transfer throughout pregnancy [97].

### 4.4. Clinical Relevance of Placental Morphology

Morphological anomalies in the placenta may arise due to a combination of genetic factors, maternal health conditions, and environmental influences. Genetic syndromes or chromosomal abnormalities can alter both fetal and placental development, leading to structural deviations such as accessory lobes or irregular shapes. Maternal conditions such as preeclampsia, gestational diabetes, or chronic hypertension are also significant contributors, as they disrupt normal vascular remodeling and trophoblast invasion. Environmental factors, including smoking, alcohol use, or nutritional deficiencies, can further exacerbate these abnormalities by impairing early trophoblast differentiation or inducing hypoxia. On a molecular level, imbalances in growth factors such as PlGF or dysregulated expression of angiogenic mediators such as VEGF may disrupt the formation and branching of villous trees, compounding the risks associated with placental morphological variations [98].

#### 4.4.1. Accessory Lobes: Succenturiate and Bilobed Placenta

One common morphological variation is the presence of accessory placental lobes, known as succenturiate lobes. These are smaller, separate lobes of placental tissue connected to the main placenta by blood vessels. Although accessory lobes often function similarly to the main placental mass, they can pose risks if not expelled after delivery. Retained succenturiate lobes can lead to postpartum hemorrhage or infection due to retained tissue within the uterus, which can delay uterine contraction and healing. In severe cases, retained placental tissue may require surgical removal, increasing the risk of complications for the mother [99].

A bilobed placenta, where the placenta is divided into two nearly equal lobes, poses similar risks. The blood vessels connecting the lobes are more exposed, increasing the risk of vasa previa—a condition where fetal blood vessels traverse the cervical opening unprotected, potentially leading to rupture and life-threatening fetal hemorrhage during labor. These accessory lobe formations highlight the importance of closely monitoring placental morphology, especially during ultrasound examinations, to prevent and manage complications associated with abnormal placental structures [100].

#### 4.4.2. Circumvallate and Circummarginate Placenta

Another morphological variation, the circumvallate placenta, occurs when the edges of the placenta fold back upon themselves, creating a thickened ring around the fetal side of the placental edge. This condition may restrict blood flow to parts of the placenta, reducing the effective surface area for maternal-fetal exchange. As a result, a circumvallate placenta is associated with intrauterine growth restriction (IUGR), preterm birth, and placental abruption—the premature detachment of the placenta from the uterine wall. In cases of circumvallate placenta, the abnormal edge formation can also increase the risk of bleeding during pregnancy, particularly in the third trimester, when demands on placental blood flow are highest [101]. Similarly, the circummarginate placenta presents as an abnormal thickening at the placental edge, though it generally poses less risk than the circumvallate form. However, both types may be associated with a higher incidence of placental separation, especially during delivery, which can lead to complications if portions of the placenta remain attached after birth [102].

#### 4.4.3. Placental Shape and Umbilical Cord Insertion Variations

The overall shape of the placenta and the insertion site of the umbilical cord are critical factors influencing placental function. Velamentous cord insertion, where the umbilical cord attaches to the membranes instead of the placental body, is well-documented to increase the risk of complications. These include reduced blood flow, vessel rupture, and vasa previa, which pose life-threatening risks to the fetus during labor [103]. In contrast, the implications of marginal cord insertion, where the cord attaches at the placental edge, remain more controversial. Some studies suggest that marginal insertion does not significantly impact fetal growth or pregnancy outcomes unless accompanied by other risk factors such as placental insufficiency or maternal hypertension. For instance, Siargkas et al. found no notable complications associated with marginal insertion in low-risk pregnancies [104]. However, other research, such as Wen et al., has associated marginal insertion with an increased risk of fetal growth restriction in pregnancies complicated by preeclampsia or gestational diabetes [105]. These contrasting findings highlight the importance of context in assessing the clinical relevance of marginal cord insertion.

Irregular placental shapes, such as lobed or discoid configurations, can further impact blood flow distribution. While most variations do not lead to complications, significant deviations from the typical round or oval shape may affect nutrient and oxygen delivery to the fetus. These structural variations underscore the importance of careful monitoring during pregnancy to identify potential risks early, particularly when other complicating factors are present [106].

#### 4.4.4. Implications of Morphological Variations for Long-Term Health

The impacts of abnormal placental morphology may extend beyond the pregnancy and neonatal periods, affecting the long-term health of both mother and child. For example, insufficient blood flow due to structural anomalies such as circumvallate placenta or velamentous cord insertion may lead to fetal hypoxia and low birth weight, conditions associated with an increased risk of metabolic disorders, cardiovascular disease, and neurodevelopmental delays later in life. From a maternal perspective, complications arising from retained placental tissue can predispose mothers to postpartum infections, delayed uterine recovery, and, in some cases, chronic uterine adhesions or scarring [107].

In conclusion, placental morphology plays a vital role in determining the success and health outcomes of a pregnancy. Variations in structure—whether related to accessory lobes, placental shape, or umbilical cord insertion—can introduce significant risks that require clinical awareness and appropriate management. Ultrasound imaging and other prenatal diagnostic tools are essential for early detection of these morphological anomalies, allowing healthcare providers to anticipate and address potential complications.

## 5. Discussion

The progression of placental development, beginning from implantation to the establishment of a fully functional placental-uterine interface, reflects a complex series of morphological and biochemical adaptations. This developmental process involves multiple stages, each with specific roles that prepare the placenta for efficient nutrient and gas exchange, immune tolerance, and fetal support. Each layer within the placenta—comprising the chorionic plate, basal plate, and intervillous space—plays a unique role in supporting maternal-fetal interaction [42].

To support the placental circulation, significant vascular remodeling occurs within the maternal spiral arteries. Extravillous trophoblast invasion plays a crucial role in transforming these arteries into low-resistance vessels, ensuring a steady, high volume blood supply to the intervillous spaces. This transformation is essential to prevent fluctuations in blood flow and pressure that could compromise placental function and fetal health. The remodeled spiral arteries provide a continuous supply of oxygen-rich blood, allowing the fetal capillaries within the villi to absorb oxygen and nutrients while releasing waste products into maternal circulation [25].

Understanding the intricacies of placental development and morphology is crucial for clinicians, as deviations in these processes often underpin major pregnancy complications. Studies have consistently linked abnormal trophoblast invasion and insufficient remodeling of maternal spiral arteries with conditions such as preeclampsia and intrauterine growth restriction (IUGR). Inadequate transformation of spiral arteries can lead to restricted blood flow, resulting in placental hypoxia and triggering inflammatory responses that contribute to preeclampsia. This connection underscores the necessity for clinicians to recognize early markers of abnormal placental development through imaging or biochemical markers, potentially allowing for timely intervention [72].

In addition to vascular adaptations, morphological variations in the placenta, such as succenturiate lobes or circumvallate placentas, carry significant clinical implications. These structural anomalies can lead to complications such as postpartum hemorrhage or fetal growth restriction. For instance, succenturiate lobes are accessory lobes that develop in the membranes and may cause complications such as vasa previa, postpartum hemorrhage, and retained placenta. By familiarizing themselves with these morphological variations, clinicians can better anticipate potential challenges during delivery and postpartum management [108].

The immune adaptations at the maternal-fetal interface are essential for a healthy pregnancy, as they establish an environment of tolerance while protecting against infections. The maternal immune system must balance the recognition of fetal cells, which carry paternal antigens, with the need to prevent an immune response that could harm the fetus. These adaptations occur primarily in the decidua, where a specialized immune cell environment is created to support trophoblast invasion, vascular remodeling, and fetal development. Decidual macrophages, uNK cells, and T-regulatory cells (Tregs) are pivotal players in this immune environment. Recent studies, including those by Mori et al., emphasize the importance of these immune cells in promoting tolerance. For example, uNK cells, unlike cytotoxic NK cells in other tissues, are modified to support trophoblast invasion and vascular remodeling rather than to initiate an immune attack. These uNK cells secrete cytokines and growth factors that help remodel maternal spiral arteries, ensuring sufficient blood flow to the placenta. Tregs also contribute to immune tolerance by suppressing potential maternal immune responses against fetal antigens, reducing the likelihood of immune-mediated pregnancy complications [109].

Another critical immune adaptation is the expression of human leukocyte antigen-G (HLA-G) by extravillous trophoblasts. This unique HLA variant helps trophoblasts evade immune detection, as it does not provoke a strong maternal immune response. The presence of HLA-G has been associated with a lower risk of immune-mediated pregnancy disorders, such as recurrent miscarriage and preeclampsia. As research into HLA-G and other immune-modulating molecules progresses, there is potential for new diagnostic markers that could help identify pregnancies at risk for immune-related complications early, offering clinicians opportunities for timely intervention [67].

The hemochorial nature of human placentation, where maternal blood comes into direct contact with the syncytiotrophoblast, also necessitates an effective immune adaptation strategy. Syncytiotrophoblasts lack traditional HLA molecules, minimizing the likelihood of activating maternal immune cells that might otherwise recognize and attack foreign antigens. This adaptation not only ensures immune tolerance but also creates a more stable environment for nutrient and gas exchange, protecting fetal development. These immune adaptations, therefore, are integral to the success of the placental-uterine interface, and disruptions in this balance could lead to adverse pregnancy outcomes [110].

Research increasingly shows that placental abnormalities are linked to long-term health consequences for both the mother and child. This concept, known as “fetal programming”, suggests that disruptions in the intrauterine environment can influence the development of chronic diseases later in life. Studies by Burton and Fowden highlight the impact of placental structure and function on fetal resource allocation, which in turn affects developmental pathways linked to metabolism and cardiovascular health [111].

For example, conditions such as IUGR, often associated with poor placental vascularization or reduced maternal blood flow, have been linked to a higher risk of metabolic syndrome, hypertension, and type 2 diabetes in adulthood. The placental environment influences fetal growth by regulating nutrient and oxygen delivery, and insufficient supplies during critical developmental periods can prompt the fetus to adapt its metabolism to a “thrifty phenotype”. This adaptation, while beneficial for short-term survival, may predispose the individual to metabolic disorders later in life, particularly if exposed to a nutrient-rich environment postnatally [112].

Abnormal placental morphology, such as circumvallate or succenturiate lobes, has also been associated with adverse long-term outcomes. Structural anomalies that limit nutrient delivery or disrupt maternal-fetal exchange can lead to fetal hypoxia and malnutrition, which have lasting effects on fetal organ development and function. These morphological variations may affect the endocrine function of the placenta, altering hormone levels that regulate fetal growth and immune development. Consequently, children born with IUGR, or other complications related to abnormal placentation, are more susceptible to developmental delays, immune dysfunction, and chronic diseases [81].

For the mother, abnormal placental function during pregnancy may elevate the risk of cardiovascular diseases, such as hypertension and heart disease, in later years. Preeclampsia, a condition linked to inadequate spiral artery remodeling and placental hypoxia, has been associated with a twofold increase in the risk of maternal cardiovascular disease postpartum. The inflammation and vascular changes experienced during preeclampsia may cause lasting damage to maternal blood vessels, predisposing these women to heart disease and stroke later in life [113].

The implications of abnormal placentation underscore the importance of monitoring placental health throughout pregnancy. Advanced imaging techniques and biomarkers are improving the early detection of placental abnormalities, providing clinicians with critical information to manage high-risk pregnancies proactively. Early detection and intervention can mitigate some long-term health risks by improving fetal conditions in utero, potentially reducing the likelihood of chronic diseases later in life for both mother and child [114].

This review provides a narrative summary of the current knowledge on placental development and implantation, focusing on the interplay between molecular factors and morphology. While statistical methods, such as meta-analysis, can provide quantitative insights and strengthen conclusions, they were not employed in this manuscript due to the diverse and qualitative nature of the studies reviewed. Future research should consider conducting systematic reviews or meta-analyses to further consolidate findings and provide statistical rigor to this field.

In summary, the immune adaptations at the placental-uterine interface and the morphological characteristics of the placenta play pivotal roles in determining pregnancy outcomes and long-term health. Anomalies in these areas not only increase the risk of pregnancy complications but also have profound and lasting impacts on health. Understanding these aspects of placental biology is crucial for clinicians, as it informs strategies for early detection, prevention, and intervention, ultimately contributing to improved maternal and child health outcomes across the lifespan.

## 6. Conclusions

The placenta is a complex and dynamic organ whose development is governed by intricate molecular and morphological processes. Understanding these mechanisms, from trophoblast invasion and vascular remodeling to villous maturation and immune regulation, is essential for appreciating its role in maintaining pregnancy and supporting fetal growth. This review emphasizes the critical interplay between molecular signaling pathways and structural adaptations that ensure effective maternal-fetal exchange and immune tolerance. A deeper understanding of the molecular and morphological foundations of placental development provides valuable insights into pregnancy complications such as preeclampsia and intrauterine growth restriction. By elucidating these processes, researchers and clinicians can better interpret the intricate biology of the placenta and its implications for maternal and fetal health, paving the way for improved management and outcomes in reproductive medicine.

## Figures and Tables

**Table 1:** Overview of implantation stages, key processes, formed structures, and hormonal activity. This table summarizes the main phases of implantation—pre-implantation, implantation proper, and post-implantation—highlighting critical processes, developing structures, and hormonal changes that support successful embryo implantation and early placental development.

Stage	Timing	Key Processes	Structures Formed	Hormonal Activity
Pre-implantation	Days 1–5 post-fertilization	Fertilization in the fallopian tube; the zygote divides forming a morula and then a blastocyst; endometrial preparation through hormonal changes (progesterone and estrogen) [21].	Blastocyst (trophoblast and inner cell mass); endometrial decidual cells rich in glycogen.	Increased progesterone and estrogen prepare the endometrium for implantation, promoting decidualization [23].
Implantation Proper	Days 6–7 post-fertilization	Blastocyst attachment to endometrium; trophoblast differentiation into syncytiotrophoblast and cytotrophoblast; formation of maternal-fetal interface with trophoblastic lacunae [22].	Cytotrophoblast and syncytiotrophoblast layers; trophoblastic lacunae.	Continued progesterone activity supports implantation; syncytiotrophoblasts release human chorionic gonadotropin (hCG) to maintain corpus luteum function [24].
Post-implantation	Days 8–12 post-fertilization	Complete embedding of blastocyst within endometrium; formation of early placental structures (primary villi); establishment of uteroplacental circulation; decidua differentiation [25].	Primary villi; decidua basalis, capsularis, parietalis; early uteroplacental circulation.	hCG levels increase, maintaining the corpus luteum and sustaining progesterone and estrogen production for endometrial support [26].

**Table 2:** Comprehensive overview of villous tree development in the placenta. This table summarizes the key stages of villous development, including timing, peak phases, percentage of placental volume at term, structural characteristics, and primary functions. It highlights the progression from early mesenchymal villi to terminal villi, each stage contributing uniquely to the placental architecture and maternal-fetal exchange.

Villous Type	Timing	When Maximum	% Volume at Term	Size	Characteristic Features	Primary Function
Mesenchymal Villi [56]	5 weeks–term	0 to 8 weeks	<1%	120–250 μm (<8 weeks), 60–100 μm (>8 weeks)	Primitive stroma, thick trophoblastic cover, few fetal vessels	Proliferation and growth precursor for other villous types
Immature Intermediate Villi [50]	8 weeks–term, peaks 14–20 weeks	14 to 20 weeks	5–10%	100–200 μm, up to 400 μm	Reticular stroma with fluid-filled channels, visible Hofbauer cells, limited vascularization	Growth centers for villous tree development and branching
Stem Villi [57]	8 weeks–term	Term	20–25%	150–300 μm	Fibrotic stroma, large vessels with media and adventitia, primary structural support	Provides structural support as the ‘trunk’ of a villous tree, minimal exchange
Mature Intermediate Villi [58]	Third trimester	Third trimester	25%	80–150 μm	Loose, unoriented connective tissue fibers, capillary-rich with vascular lumens < 50%	Significant role in exchange, structural support for terminal villi formation
Terminal Villi [3]	Third trimester	Term	40–50%	60 μm	High capillary density (>50% vascular lumen), thin trophoblastic cover, main exchange site	Primary site of feto-maternal exchange due to high capillary volume and efficiency
